# Efficacy and safety of super pulse CO2 laser-assisted punctoplasty with canalicular curettage in primary canaliculitis

**DOI:** 10.1007/s10103-023-03735-z

**Published:** 2023-02-18

**Authors:** Weifeng Huang, Shujuan Cao, Lingling Xie, Xingyi Li, Ziwei Meng, Xinyue Yu, Danping Huang, Rongxin Chen, Xuanwei Liang

**Affiliations:** 1 State Key Laboratory of Ophthalmology, Zhongshan Ophthalmic Center, Sun Yat-sen University, Guangdong Provincial Key Laboratory of Ophthalmology and Visual Science, Guangdong Provincial Clinical Research Center for Ocular Diseases, Guangzhou, 510060 China; 2 Ophthalmologic Center, Affiliated Kashi Hospital of Sun Yat-sen University, First People’s Hospital of Kashi Prefecture, Kashi, 844000 China

**Keywords:** Canaliculitis, Super pulse CO_2_ laser, Punctoplasty, Minimally invasive treatment

## Abstract

The objective of this study was to evaluate the efficacy and safety of super pulse CO_2_ laser-assisted punctoplasty with canalicular curettage in primary canaliculitis. In this retrospective serial case study, the clinical data of 26 patients who underwent super pulse CO_2_ laser-assisted punctoplasty for the treatment of canaliculitis were collected from January 2020 to May 2022. The clinical presentation, intraoperative and microbiologic findings, surgical pain severity, postoperative outcome, and complications were studied. Of the 26 patients, most were females (female:male 20:6), with a mean age of 60.1 ± 16.1 years (range, 19–93). Mucopurulent discharge (96.2%), eyelid redness and swelling (53.8%), and epiphora (38.5%) were the most common presentations. During the surgery, concretions were present in 73.1% (19/26) of the patients. The surgical pain severity scores ranged from 1 to 5, according to the visual analog scale, with a mean score of 3.2 ± 0.8. This procedure resulted in complete resolution in 22 (84.6%) patients and significant improvement in 2 (7.7%) patients, and 2 (7.7%) patients required additional lacrimal surgery with a mean follow-up time of 10.9 ± 3.7 months. The surgical procedure of super pulse CO_2_ laser-assisted punctoplasty followed by curettage appears to be a safe, effective, minimally invasive, and well-tolerated treatment for primary canaliculitis.

## Introduction

Lacrimal canaliculitis is an infection of the lacrimal duct system. The major clinical signs of canaliculitis include persistent punctal discharge, punctal or canalicular swelling, tearing, and erythema in the medial canthus.

Due to its atypical clinical features in the early stages and low incidence, lacrimal canaliculitis can be somewhat difficult to diagnose and eradicate [1, 2]. It tends to be misdiagnosed as conjunctivitis, blepharitis, chalazion, dacryocystitis, etc., preventing effective treatment and therefore posing challenges for both patients and clinicians [3].

The treatments for canaliculitis include conservative and surgical management strategies. Conservative management strategies, such as irrigation with topical antibiotics, punctal dilation, manual expression, or microcurettage, may provide temporary relief. However, persistent disease and frequent recurrence are common [4–6]. Some surgical management strategies, such as canaliculotomy [7], punctoplasty, and canalicular curettage with possible stenting [8], have been shown to yield relatively good success rates. The canaliculotomy procedure, however, is more invasive, causing more damage to the canaliculus and potentially causing canalicular lumen narrowing or scarring, affecting lacrimal pump function [9]. To minimize damage to the punctum and canaliculus and achieve better surgical results, many innovative methods have been proposed, such as 4-snip punctoplasty [10], 3-snip punctoplasty [11], 2-snip punctoplasty [12], and 1-snip punctoplasty [13], as well as reconstruction of the anatomic structure of the canaliculus with stents [8]. Even so, recurrent or persistent disease remains challenging for clinicians. Therefore, more ideal management should be used to thoroughly remove canalicular concretions, cause less disruption, and maintain the postoperative drainage function of the lacrimal canaliculus in the long term. One of the key factors affecting the final result of punctoplasty is the formation and function of the punctum. Stenosis of the newly formed punctum, which results from the adjacent raw cut edges healing too quickly, always leads to persistence or recurrence of the disease.

Studies have shown that fewer myofibroblasts appeared in wounds treated with a CO_2_ laser than in surgical incisions, and they also appeared later, possibly explaining the minimal contractions in CO_2_ laser excision wounds [14]. Compared to traditional surgery, super pulse CO2 laser surgery has been found to be a safe and effective method for treating benign peri-punctal eyelid tumors [15]. Therefore, it may also be used to treat canaliculitis. To the best of the authors’ knowledge, no research has been done on this topic. CO_2_ laser surgery is an interesting technique because of its increasing range of clinical indications, its relatively low invasiveness, and its execution in an outpatient setting.

In this study, super pulse CO_2_ laser-assisted punctoplasty with canalicular curettage was performed on patients with primary canaliculitis in an outpatient setting. The clinical presentation, intraoperative and microbiologic findings, surgical pain severity, postoperative outcome, and complications were studied.

## Materials and methods

### Ethics statement

This study was approved by the institutional review board (IRB) of ZhongShan Ophthalmic Center, Sun Yat-sen University, Guangzhou, China (No. 2021KYPJ100) and adhered to the tenets of the Declaration of Helsinki. All patients provided their informed consent.

### Participants

All patients who underwent super pulse CO_2_ laser-assisted punctoplasty for the treatment of canaliculitis at Zhongshan Ophthalmic Center, Sun Yat-sen University of China, from January 2020 to May 2022 were included in this study. The diagnosis of canaliculitis was established by persistent mucopurulent discharge, or epiphora, accompanied by the presence of expressible discharge from the punctum, concretions extruding from the punctum, and swelling and erythema of the affected punctum. Prior to making the diagnosis, the puncta, eyelids, canthi, and conjunctiva were examined thoroughly. All participants underwent an 80-MHz ultrasound biomicroscopy (UBM) examination (SW-3200 L; Tianjin Suowei Electronic Technology Co., Tianjin, China) and lacrimal irrigation prior to the operation. In addition, patients with secondary canaliculitis, such as punctual plug-induced canaliculitis, and lower eyelid malpositioning, including ectropion or entropion, were excluded.

### Surgical technique of super pulse CO_2_ laser-assisted punctoplasty

**Fig. 1:**
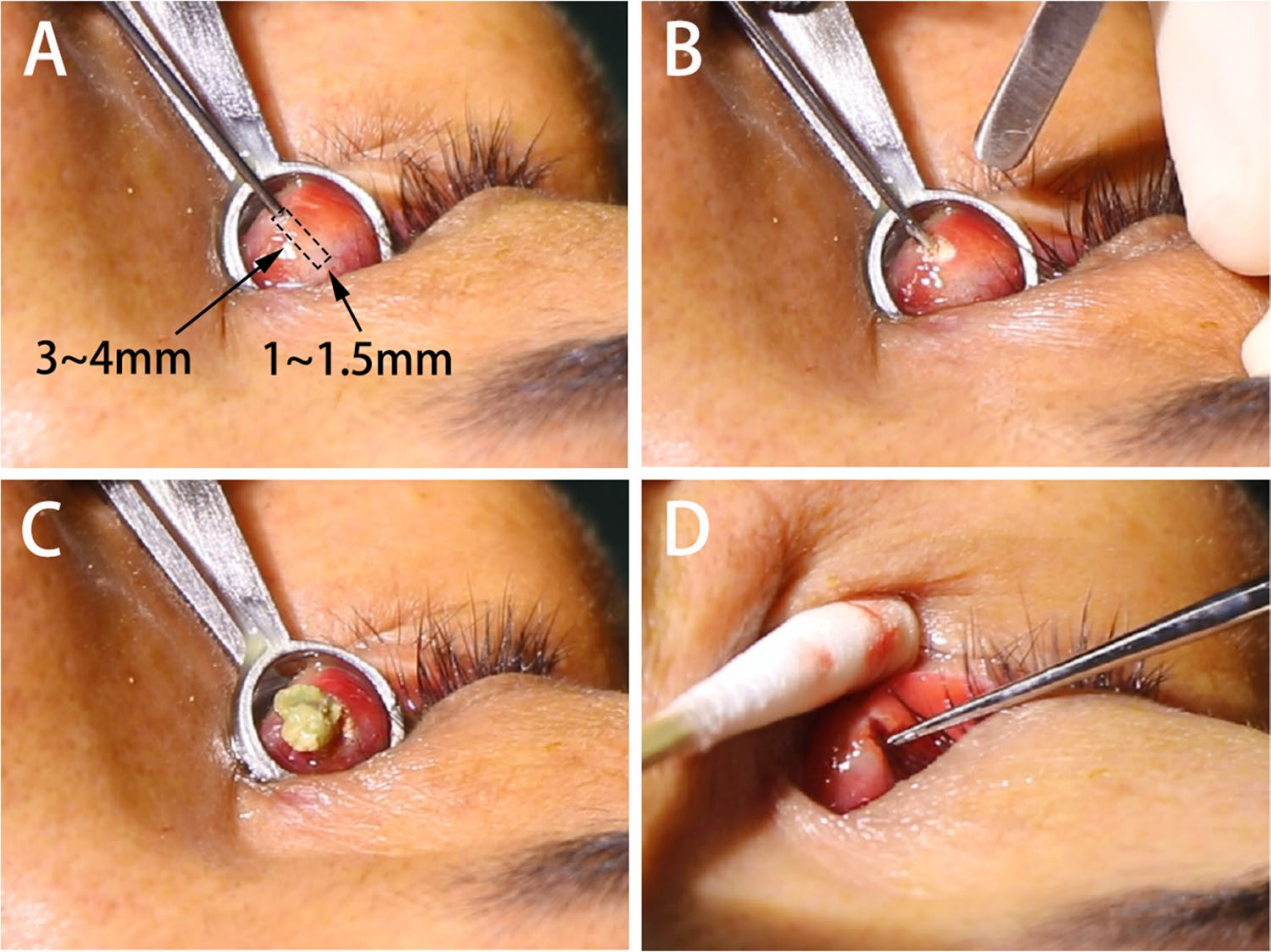
Treatment procedure for super pulse CO2 laser-assisted punctoplasty with canalicular curettage. **A** Chalazion clamp used to expose the punctum. Lacrimal duct probe inserted through the punctum. **B** Affected punctum incised along the posterior wall vertically by the laser. **C** Concretions in the canaliculus evacuated from the wound. **D** Chalazion curette inserted into the canaliculus to remove the rest concretions or granulation tissues

All procedures were performed by the same surgeon and under local infiltrative anesthesia with 2% lidocaine and with the use of a CO_2_ laser (Lumenis, Ltd., Silicon Valley, CA, USA). The patients wore protective eyewear, and the operator wore laser-protective goggles during the treatment. First, the position of the lacrimal punctum was exposed using a chalazion clamp, and then a No. 7–8 lacrimal duct probe was placed through it. Along with the lacrimal duct probe, the posterior wall of the punctum was cut vertically for 3–4 mm length and 1–1.5 mm width by the CO_2_ laser (175 mJ; spot size, 1 mm). Next, a small chalazion curette was inserted through the punctum into the canaliculus. Any concretions or granulation tissues as well as mucoid debris were removed and sent for microculture and histologic examination if possible. Lacrimal system irrigation was performed several times to ensure the absence of any residual concretions or debris (Fig. 1).

### Assessment of surgical pain severity

Pain levels were assessed by the visual analog scale (VAS), which consists of a horizontal line (10 cm long) with anchor points of “no pain” and “worst possible pain” [16]. Postoperative patients were asked to mark the line describing their pain severity during surgery.

### Postoperative care and follow-up

After surgery, all patients were treated with levofloxacin eye drops (5 ml: 24.4 mg, Zhongshan Wanhan Pharmaceutical Co., Ltd., China) 4 times daily for 2 weeks. The following data were collected: patient demographics, symptoms, intraoperative findings, microbiologic and histologic findings, postoperative UBM findings, and procedural outcome at 1 month, 3 months, and 6 months after surgery.

### Assessment of surgical outcome

The surgical outcome of this study was determined subjectively according to the strict criteria offered by Moore et al. [17]. The patients were asked to rate their pre- and post-operative complaints on a 5-point Likert scale, as follows: (1) cure and no complaints of mucous discharge or epiphora; (2) significant improvement (symptoms are at least 80% better); (3) moderate/slight improvement; (4) unchanged; and (5) worsening of the symptoms. Points 1 and 2 were considered to indicate a “satisfactory” outcome, while the other points were considered to indicate an “unsatisfactory” outcome.

## Results

### Primary canaliculitis mainly involved the unilateral lower lacrimal canaliculus in middle-aged and older women

Twenty-six eligible primary canaliculitis patients who underwent laser-assisted punctoplasty were reviewed. There were 20 (77%) females and 6 (23%) males, with a mean age of 60.1 ± 16.1 years (range, 19–93). All but 1 patient presented with unilateral eye involvement. Most patients (15 patients) showed involvement of the lower canaliculus, followed by 6 patients with involvement of the upper canaliculus and 5 patients with involvement of both the lower and upper canaliculi. The left eye was involved in 13 patients (50%), while the right eye was involved in 12 patients (46.2%), and the lower canaliculi of both eyes were involved in 1 patient (3.8%). The mean symptom duration was 38.0 ± 44.1 months (range, 1 month to 15 years).

### Most patients presented with mucopurulent discharge, as well as lacrimal patency

The most common presentation was mucopurulent discharge (25 patients; 96.2%), followed by eyelid redness and swelling (14 patients; 53.8%) and epiphora (38.5%). Most patients (24 patients; 92.3%) had expressible discharge from the punctum. The other clinical signs were pouting punctum (16 patients; 61.5%), punctal swelling and erythema (22 patients; 84.6%), and thickening of the canalicular portion of the eyelid margin (14 patients; 53.8%). Seventeen patients (65.4%) had a patent lacrimal drainage system, and 9 patients (including 2 with canalicular obstruction, 4 with lacrimal duct stenosis, and 3 with total nasolacrimal duct obstruction) had pathologic lacrimal characteristics as determined by lacrimal syringing examination (Table 1).

**Table 1:** Clinical presentations of primary canaliculitis patients

Clinical presentations	No. (%)
Presenting symptoms	
Mucopurulent discharge	25 (96.2%)
Epiphora	10 (38.5%)
Eyelid redness and swelling	14 (53.8%)
Conjunctival injection	
Clinical signs	
Discharge from punctum	24 (92.3%)
Pouting punctum	16 (61.5%)
Punctal swelling and erythema	22 (84.6%)
Thickening of canalicular portion of eyelid margin	14 (53.8%)
Syringing	
Patent	17 (65.4%)
Canalicular obstruction	2 (7.7%)
Lacrimal duct stenosis	4 (15.4%)
Nasolacrimal duct obstruction	3 (11.5%)

### The most common combinations of pathogens were Actinomyces and Streptococcus species

**Fig. 2:**
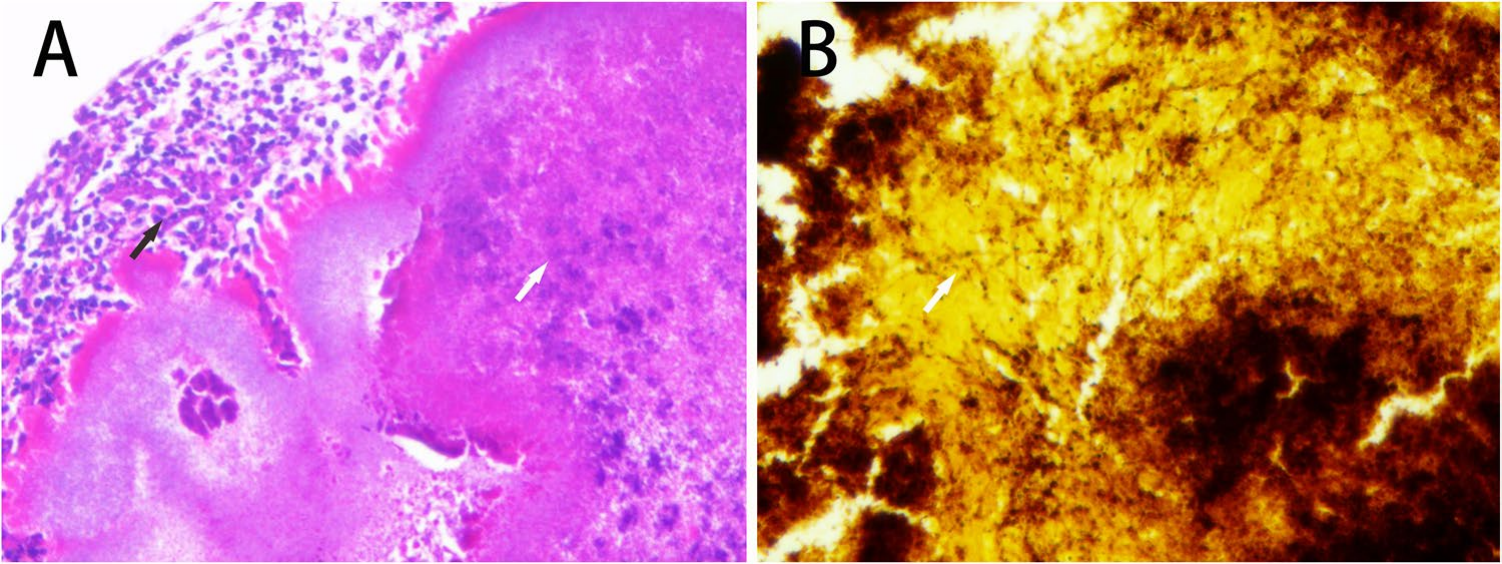
**A** The acute and chronic inflammatory cell (black arrow) cuff was frequently conspicuous around the bacterial concretions (white arrow) (haematoxylin–eosin, × 200). **B** Hyphal fungal forms (white arrow) were prominent in the concretions (Gomori methenamine silver, × 400)

The contents of the canaliculus were easily evacuated in all patients via the posterior wall punctal incision. Culture of the discharge was performed in 22 (23 eyes) of the 26 patients (84.6%). Microbiologic results were positive in 12 (13 eyes) of the 22 patients (54.5%), and the common species included *Streptococcus* species in 4 patients (17.4%) and *Staphylococcus* species in 5 patients (21.7%). Sulfur granules or concretions were removed from 19 patients and sent to the pathology laboratory for histologic examination. *Actinomyces* species were identified in all 19 patients’ concretions by Gomori methenamine silver staining during histologic examination (Fig. 2).

### Super pulse CO2 laser-assisted punctoplasty was a safe, well-tolerated, and effective treatment for primary canaliculitis

The pain severity during surgery ranged from 1 to 5 according to the VAS, with a mean score of 3.2 ± 0.8. The postoperative follow-up period lasted between 3 and 17 months, with a mean time of 10.9 ± 3.7. The subjective assessment of the study group demonstrated cure in 22 patients (84.6%), significant improvement in 2 patients (7.7%), and moderate/slight improvement in 2 patients (7.7%). Therefore, the surgical outcome of 24 patients (92.3%) was subjectively determined to be satisfactory, while that of 2 patients (7.7%) was subjectively determined to be unsatisfactory (Table 2).

**Table 2:** Treatment outcomes and VAS scores after surgery

	No. (%)	Notes
Outcome		
Cure	22 (84.6%)	
Significant improvement	2 (7.7%)	Preexisting canalicular obstruction in 1 patient and upper punctal stenosis in the other patient
Moderate/slight improvement	2 (7.7%)	Preexisting nasolacrimal duct pathologic features on both patients
Unchanged	0	
Worsening	0	
VAS score		
1	1 (3.8%)	
2	2 (7.7%)	
3	16 (61.5%)	
4	7 (26.9%)	
5	1 (3.8%)	

Among the patients with significant improvement, one patient was noted to have preexisting lower canalicular obstruction before surgery, and the other patient was found to have upper punctal stenosis 1 month after surgery, secondary to the healing of the adjacent raw cut edges. Both of these patients only felt moisture in the operated eye without epiphora or mucous discharge and did not need further treatment. However, the remaining 2 patients who had preexisting pathologic nasolacrimal duct features before surgery showed persistent signs of canaliculitis to varying degrees. Additional lacrimal surgery was recommended. One of the patients accepted further treatment and achieved complete resolution after dacryocystorhinostomy. The other patient declined further treatment.

### The punctum healed smoothly postoperatively, and a near-normal canalicular lumen was achieved over time

**Fig. 3:**
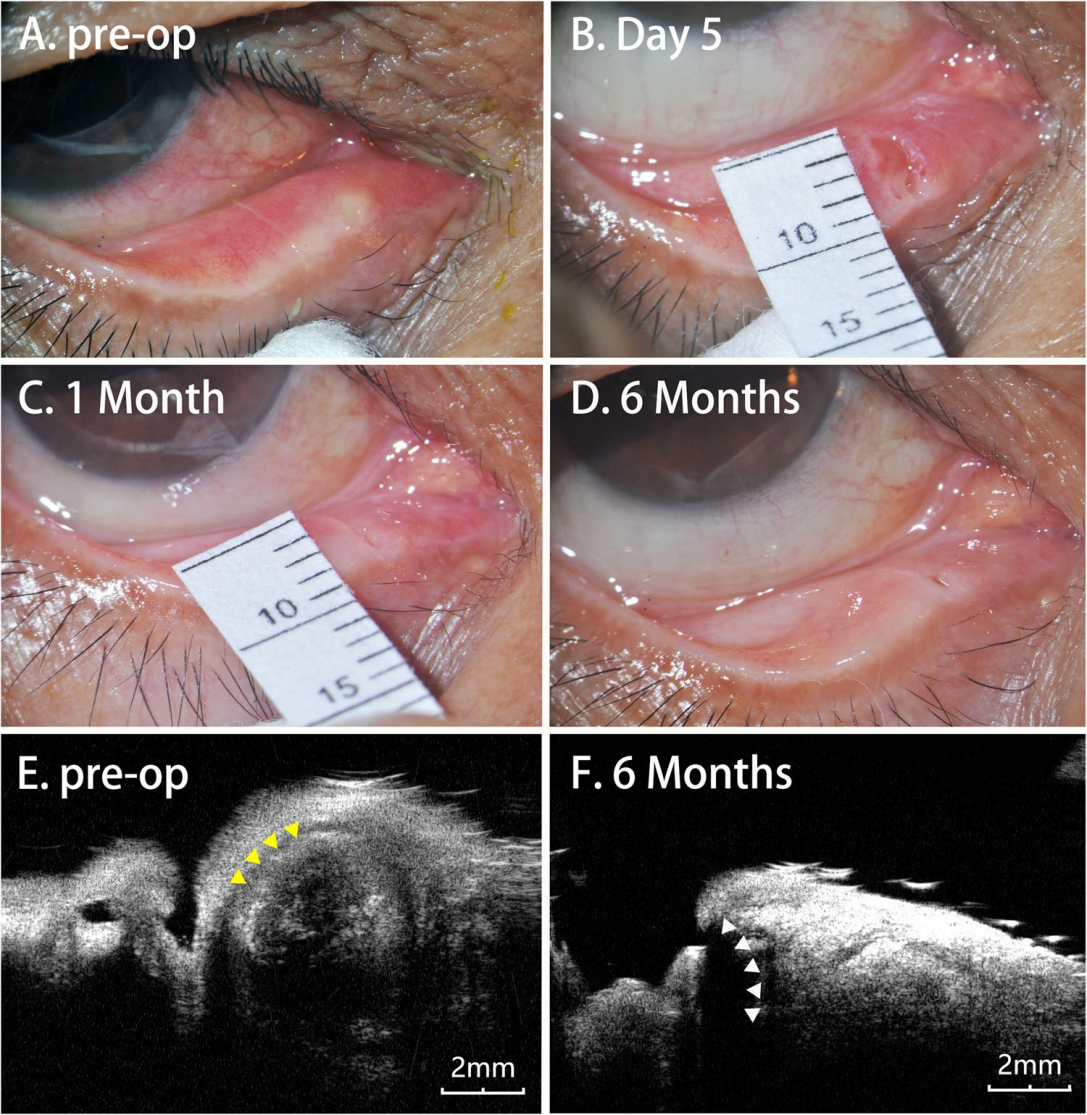
Preoperative and postoperative changes in the wound and UBM findings. **A** Mucopurulent discharge from the puncture was noted preoperatively. **B** Postoperative day 5, a 4-mm wound created by the CO2 laser was seen. **C** One month postoperatively, the wound was found to be healing smoothly with the adjacent epithelium to form a new punctum approximately 3 mm in length. **D** Six months postoperatively, the new punctum eventually stabilized in the form of a crack. **E** Preoperative UBM revealed the lower ectatic canaliculus near the punctum and thickened lumen wall (yellow arrowheads) accompanied by highly reflective intracanalicular concretions indicated by ultrasonic shadow. **F** Postoperative UBM showed the size of the canalicular lumen (white arrowheads) near the punctum at the follow-up visit 6 months after surgery

During the recovery process, we noted that the wound created by the CO_2_ laser healed smoothly with the adjacent epithelium to form a new punctum at approximately 1 month after surgery, accompanied by slight contraction in the following 2 months and gradual stabilization. Preoperative UBM examination (vertical section) showed a thickened lumen wall and obvious ectasia of the lacrimal canalicular lumen in all lacrimal canaliculitis patients, especially patients with intracanalicular concretions. Six months after surgery, postoperative UBM examination of the patients with concretions showed that the canalicular lumen had become significantly smaller and regained an anatomic structure very similar to the normal structure, although it was still larger than the normal size (Fig. 3).

## Discussion

There is a high rate of delayed or incorrect diagnosis due to the low incidence and lack of typical clinical features of primary canaliculitis. Similar to other studies [3, 18, 19], in this study, the most common symptom was mucopurulent discharge (96.2%), and the most common sign was discharge expression from a pressed punctum. According to our results and other previous research [3, 13], this condition often affects the lower canaliculus unilaterally and is more common in women. This female predominance may reflect hormonal or physiologic changes in women during menopause, which result in a decrease in tear production and cause the breakdown of the protective barrier against infections [20].

*Actinomyces*, thought to be the most common pathogenic bacterium in canaliculitis, is a cast-forming gram-positive anaerobe that is very difficult to grow in culture [7, 21, 22]. It is always related to the pathologic hallmark of sulfur granules and can be sensitively identified by pathologic laboratory examination. Our results showed sulfur granules or concretions in 19 of the 26 patients, and the subsequent histologic staining revealed *Actinomyces* species in all 19 patients, which is consistent with the research reported by Repp et al. [21]. However, it is worth noting that these species can also be seen in nocardiosis, chromomycosis, and botryomycosis, as previously reported [13]. In addition, canaliculitis can be caused by polymicrobial infections, including those caused by *Streptococcus*, *Staphylococcus*, and *Propionibacterium* species [19]. We observed that the predominant bacterial species isolated from the cultures were *Staphylococcus* (5/13, 38.5%) and *Streptococcus* (4/13, 30.8%), similar to those found in some prior studies [3, 4]. Perry et al. [23] suggested that *Actinomyces* is the predominant organism of the concrements, and gram-positive cocci frequently colonized the outer edges, contributing to the purulent nature of canaliculitis.

The exact pathogenesis of canaliculitis is still unknown. Previous studies have shown that the presence of a diverticulum or any obstruction of the lacrimal system, resulting in a stagnation in tear flow and drainage, may lead to an accumulation of bacteria in the proximal lacrimal system, thus increasing the risk of canaliculitis [4, 6, 24]. Take plug-related canaliculitis as an example. The prevalence of canaliculitis was 24% per patient and 16.6% per SmartPlug after SmartPlug insertion in a previous study. The median time from SmartPlug insertion to the onset of canaliculitis was 4.7 (1.4–6.0) years [25], a finding that is similar to the results of a study by Huang et al. [26], in which the average time interval was 5.5 years. In this sense, it is understandable that obstruction of the lacrimal system can cause tear flow stagnation and is a risk factor for canaliculitis formation.

Generally, it takes several years, not several days or months, to develop canaliculitis after the use of lacrimal plugs. However, in our study, a 22-year-old female patient with two puncta in the lower canaliculus of the left eye developed canaliculitis within 1 month. She complained of an increase in mucous discharge after syringing during the preoperative preparation for cataract surgery, and she had never encountered a similar situation before. The lacrimal drainage system was patent in this case, and during the treatment, we also found the existence of concretions. For this reason, we suspected that mucosal injury caused by syringing or other elements, including lacrimal plugs, may be another important predisposing factor. Further research is needed to prove this hypothesis.

Due to the excellent exposure afforded to the surgeon and the thorough purge that follows, canaliculotomy with curettage has also been shown to have good results. However, excision of any part of the canaliculus will compromise the puncto-canalicular function of the lacrimal pump, and it has been indicated that 4 of 20 patients develop postoperative epiphora with a patent lacrimal system after canaliculotomy [6]. To avoid these complications, Khu and Mancini [27] reported a modification to the canaliculotomy technique; the modified technique began 2 mm from the punctum and was accompanied by curettage followed by Mini-Monoka stent insertion to prevent canalicular stricture. Su et al. [8] reconstructed the anatomic structure of the canaliculus after canaliculotomy via canaliculoplasty with a silicone stent. These procedures appear to be an encouraging treatment for canaliculitis, but the risk of postoperative epiphora and canalicular scarring is still unknown. In addition, stent placement requires additional operative time and expense.

In this study, the application of a super pulse CO_2_ laser in the treatment of canaliculitis is presented for the first time. It has been proven to be a highly effective and well-tolerated alternative therapy. The CO_2_ laser is recognized as an ideal surgical laser and has been widely used for both cosmetic and therapeutic purposes. When compared to other lasers in incisional and ablative surgery, the CO_2_ laser shows considerable advantages, such as minimal bleeding with optimal visibility and deeper noncontact cuts [28]. Absorption of the laser light in different emission modes will produce different photothermal effects, such as coagulation, vapourization, and photoablation. Consequently, it can be used for various procedures, such as incisions, excisions, vapourizations, and coagulations, with great precision. A previous study demonstrated that the super pulse laser emission mode produced less lateral thermal damage than the continuous wave (CW) laser emission mode; additionally, lower laser powers created less thermal damage [29, 30]. CO_2_ laser excision wounds tend to heal with a minimal degree of contraction because of the lower number and later appearance of myofibroblasts in these wounds than in surgical incisions [14]. Because of the precision of the laser, the wound edge and nearby necrosis are much more regular, a less severe inflammatory reaction is induced, and a better cosmetic outcome is achieved [31].

Therefore, we applied a super pulse CO_2_ laser to create an expanded opening via vertical canaliculotomy, allowing for the successful removal of the canalicular contents while minimizing trauma and maximizing the preservation of the anatomy and physiologic functioning of the lacrimal system. This technique can be largely avoided the recurrence of the disease secondary to the quick healing of the adjacent raw cut edges made by micro-scissors. It has been proven to have a high success rate, does not require stents, is associated with fewer cosmetic complications, and is more comfortable for the patient. Twenty-four (92.3%) of the 26 patients achieved subjectively satisfactory outcomes, and no further surgical treatment was needed. Only 2 patients who were noted to have a preexisting obstruction of the nasolacrimal duct before surgery required further treatment. CO_2_ laser surgery also offers a high level of patient comfort because it does not require primary wound closure with sutures or stent insertion, which is consistent with our VAS score results (mean ± standard deviation, 3.2 ± 0.8). The ectatic canalicular lumen induced by the large concretions was significantly smaller postoperatively, as demonstrated by UBM examination. In addition, we observed preexisting canalicular obstruction in some patients preoperatively, and the symptoms and signs of canaliculitis disappeared after surgery even though the canalicular obstruction still existed. The characteristic of these patients was that they had upper or lower canalicular involvement only, and the other canaliculus was patent.

As a retrospective study, this study is limited by a small sample size and a somewhat limited follow-up period. Direct comparison with other techniques and studies is limited. At the follow-up examination, patients without canaliculitis were not routinely treated with syringes unless recurrence or other complications occurred. Therefore, it is possible that there were more patients with canalicular obstruction who were asymptomatic. In conclusion, the surgical procedure of super pulse CO_2_ laser-assisted punctoplasty followed by curettage appears to be a safe, simple, effective, minimally invasive, and well-tolerated treatment for primary canaliculitis in an outpatient setting. Additionally, it has a relatively short learning curve, and a very short time is needed for clinicians performing this surgery to achieve proficiency. The advantages of this technique include reduced surgical waiting lists, resources and durations, and suitability for patients who fear surgery or are elderly. Furthermore, as no sutures or stents are placed, there is no need for frequent follow-ups.
